# Identification of genes and pathways involved in malignant pleural mesothelioma using bioinformatics methods

**DOI:** 10.1186/s12920-021-00954-7

**Published:** 2021-04-13

**Authors:** Xingsheng Liu, Kun Qian, Gaojun Lu, Peng Chen, Yi Zhang

**Affiliations:** Department of Thoracic Surgery, Xuanwu Hospital, Capital Medical University, 45 Changchun Street, Beijing, 100053 China

**Keywords:** Microarray, Gene expression profile, Cancer, Differentially expressed genes, Protein–protein interaction, Bioinformatics

## Abstract

**Background:**

Malignant pleural mesothelioma (MPM) is a rare tumor in the pleura. This study was carried out to identify key genes and pathways that may be involved in MPM.

**Methods:**

Microarray datasets GSE51024 and GSE2549 were analyzed for differentially expressed genes (DEGs) between normal and MPM tissues. The identified DEGs were subjected to functional analyses using bioinformatics tools.

**Results:**

A total of 276 DEGs were identified, consisting of 187 downregulated and 79 upregulated genes. Gene ontology and Kyoto encyclopedia of genes and genomes pathway enrichment analysis indicated that the DEGs were enriched in extracellular structure organization, extracellular matrix, and ECM−receptor interaction. Due to high degree of connectivity among 24 hub genes, EZH2 and HMMR are likely to play roles in the carcinogenesis and progression of MPM. The two genes were found over-expressed in MPM tissues. Patients with elevated EZH2 and HMMR expressions had poor overall survival.

**Conclusions:**

EZH2 and HMMR are identified to be the hub genes for MPM and they may be further characterized to better understand the molecular mechanisms underlying the carcinogenesis of MPM.

## Background

Malignant pleural mesothelioma (MPM) is a rare and highly lethal neoplasm arising from the mesothelial cells lining the pleural cavity [1]. However, due to wide use of asbestos, its incidence has been dramatically increasing since the mid-twentieth century [2]. Among histological types (epithelial, sarcomatoid, and biphasic or mixed), the sarcomatoid carcinoma is particularly associated with a more aggressive progression [3]. Because of a lack of effective treatment of the disease, overall survival (OS) of patients with MPM is around one year after diagnosis and up to two years after receiving intensive multimodality therapy [4]. Therefore, it is important to elucidate the molecular mechanisms underlying the carcinogenesis and progression of MPM.

During the past decade, tremendous progress has been made in elucidating the pathogenesis of MPM. For example, microarray technology has been used to examine the oncogenic genetic alterations in MPM, such as homozygous deletion of the 9p21 locus which harbors the p16/CDNK2A gene, germline mutations of BAP1 and mutations of multiple Hippo genes [5, 6]. Moreover, NAT2, a polymorphic gene encoding enzymes in xenobiotic and oxidative metabolism or involved in genome stability, is found to increase MPM risk in asbestos-exposed populations [7]. From epigenetic perspectives, SETD2 (SET domain containing protein 2, a histone methyltransferase), specifically H3K36me3 (histone H3 trimethylated at lysine 36), is identified as epigenetic regulator of gene expression associated with MPM [5]. Based on the methylation status of the human androgen receptor gene, MPM was found to have a polyclonal origin [8]. An exploratory analysis proposed that PI3K/Akt/mTOR signaling pathways and downstream proteins are frequently activated in MPM and can be used to provide prognostic information [9]. In addition, chronic inflammation also plays a key role in the pathogenesis of MPM as a result of asbestos exposure [10]. However, the moclular mechanisms underlying the disease are still largely unclear and more genes need to be identified to advance our understanding.

In the present study, two mRNA microarray datasets from Gene Expression Omnibus (GEO) databases were analyzed to identify the common DEGs between MPM and non-cancerous tissues. Enrichment analysis, network-based approaches and ONCOMINE data mining were applied to identify hub genes related to MPM. The findings would provide new insights into moclular mechanisms related to MPM pathogenesis and clues to develop diagnostic and therapeutic approaches for the caner.

## Materials and methods

### Microarray data

The mRNA expression profiles were obtained from the GSE2549 and GSE51024 datasets [11]. The GSE2549 dataset contains 40 MPM samples and 4 non-cancerous tissue samples. The GSE51024 dataset has 55 MPM samples and 41 non-cancerous tissue samples (Table 1). The two datasets were downloaded from GEO (http://www.ncbi.nlm.nih.gov/geo) which is a public repository containing data obtained through high throughout gene expression assays, chips and microarrays. The data were uniformly pre-processed using the Robust Multichip Average algorithm for background correction, quantile normalization and log2-transformation [12]. The probes were converted into corresponding genes using the annotation information available from the gene platforms (GPL96-57554 and GPL570-55999, respectively.).

**Table 1:** Details of MPM mRNA expression data collected from GEO data sets

Author (year)	Materials	Accession/ID	Platform	Normal samples	MPM samples
	Tissue	GSE2549	GPL96-57554	4	40
Suraokar et al. [11]	Tissue	GSE74190	GPL570-55999	41	55

*GEO* Gene Expression Omnibus, *GPL* GEO platform, *MPM* Malignant Pleural Mesothelioma, *mRNA* messenger RNA

### Identification of differentially expressed genes (DEGs)

The DEGs between MPM and non-cancerous tissue samples were identified using the limma package in R language. Adjusted P-values (adj. P) and false discovery rate were used to balance statistically significant genes and false-positives. The threshold of adj. P was set at < 0.05 and | log2 (fold-change) |> 1. Volcano figures were plotted to identify the DEGs in the two datasets using ggplot2 package (version 3.5.3). Venn diagram was then constructed to determine the common DEGs originated from both datasets.

### Construction of protein–protein interaction (PPI) network, module analysis and hub gene selection

The Search Tool for the Retrieval of Interacting Genes (STRING; http://string-db.org) (version 10.0) provides uniquely comprehensive interaction information of experimental and predicted proteins. In this study, the PPI network of common DEGs was constructed using STRING online database. A combined score > 0.4 was considered as the reliability threshold for interaction. Cytoscape (version 3.4.0; www.cytoscape.org), an open source bioinformatics software platform, was used to visualize the molecular interactions in the PPI networks obtained with the STRING online databases. Subsequently, the module analysis of the PPI network was preformed using Molecular Complex Detection (MCODE) (version 1.5.1), which is an APP for analyzing hub genes according to topology to find highly connected regions which had MCODE scores > 5, degree cut-off = 2, node score cut-off = 0.2, Max depth = 100 and k-score = 2. Then, the hub genes were selected from the module based on both the degree of the connectivity and the node status.

### Gene ontology (GO) and Kyoto encyclopedia of genes and genomes (KEGG) pathway enrichment analysis

GO is widely used to annotate genes and gene products, and to functionally characterize high-throughput genome or transcriptome data. KEGG are databases capable of interacting with genomes, biological pathways, diseases, drugs, and chemicals [13, 14]. GO and KEGG pathway enrichment analyses were carried out using clusterProfiler package and Signaling Pathway Impact Analysis (SPIA) (v3.4.0). The threshold was set at *P*-value < 0.05. Enriched GO terms or KEGG signaling pathways were ranked based on the degree of connectivity of hub genes using GOplot package (version 1.4.0).

### Validation of the selected hub genes in bioinformatic database

Oncomine (www.oncomine.org; Ion Torrent; Thermo Fisher Scientific, Inc.) is an online cancer microarray database developed to facilitate the discovery of oncogenes through genome-wide expression analyses [15]. To validate the expression level of the identified hub genes in MPM, gene expression data in the Oncomine database were analyzed with *P*-value set to < 0.05, thresholds for fold-change and gene rank set to ‘all’. In order to calculate the prognostic significance of the selected hub genes, Kaplan–Meier survival analyses were performed based on the clinical information from TCGA datasets using UALCAN, which is a comprehensive, user-friendly, and interactive web resource [16]. For this analysis, the patients were separated into high and low expression groups according to the median of the hub gene expression levels.

## Results

### Identification of DEGs

**Fig. 1:**
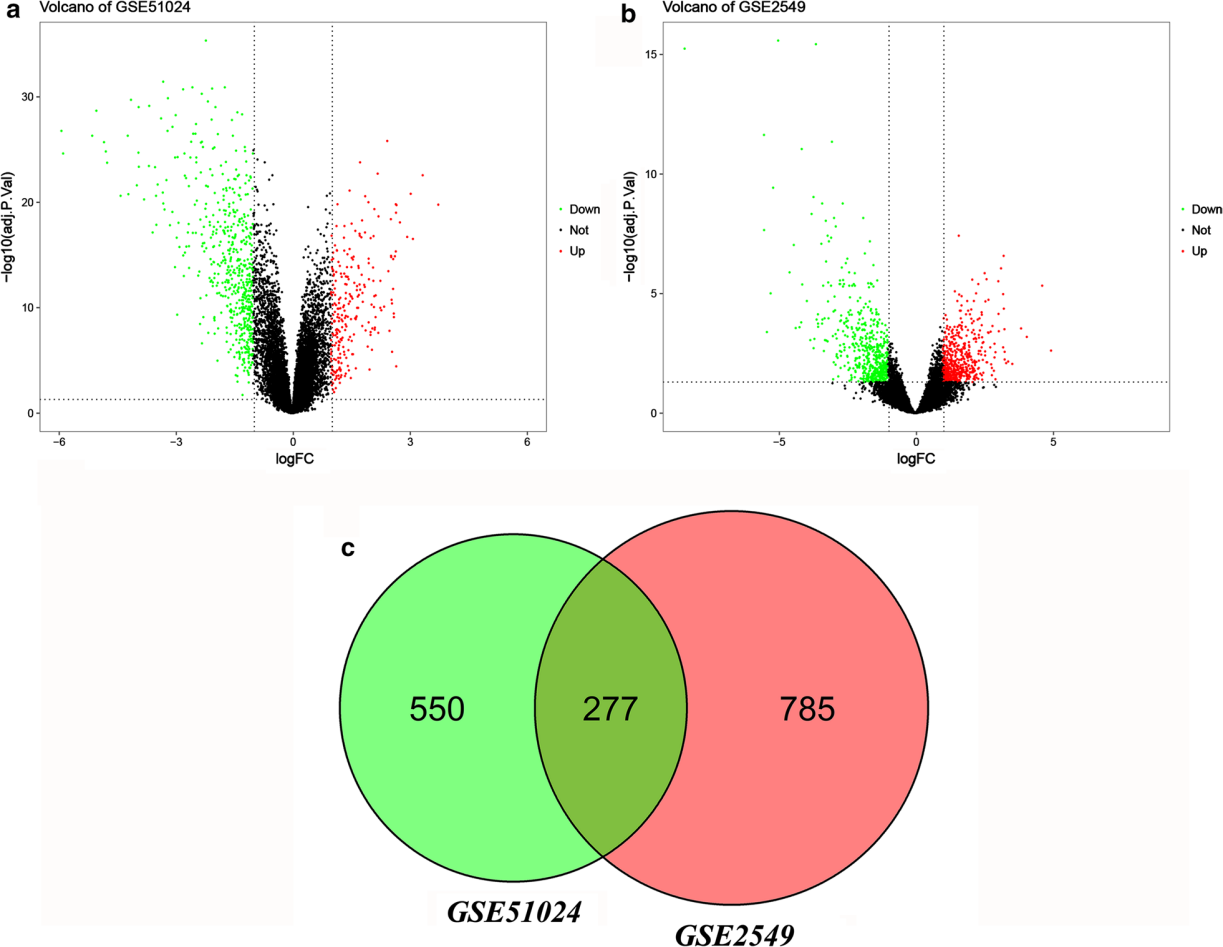
Volcano plot and Venn diagram of DEGs in MPM mRNA expression profiling datasets. Volcano plots of the gene expression data from the **a** GSE50124, **b** GSE2549 datasets. DEGs were selected by P < 0.05 and |log2 (fold-change)|> 1.The horizontal axis represents the log2 (fold change) between MPM samples and non-cancerous tissue samples; the vertical axis represents the -log10 (adjusted P-values). The red dots represent the co-upregulated DEGs and the blue dots represent the co-downregulated DEGs. **c** The grey overlap represents the common DEGs between the two datasets. *DEGs* differentially expressed genes

After standardization of the microarray results, a total of 827 and 1062 DEGs were identified in the GSE51024 and GSE2549 datasets, respectively. The volcano figures were ploted to visualize DEGs between MPM and non-cancerous tissue samples (Fig. 1a, b). The Venn diagram showed that 276 DEGs were common in the two datasets, of which 187 and 79 genes were downregulated and upregulated in both datasets, respectively (Fig. 1c).

### Construction of PPI network and module analysis

**Fig. 2:**
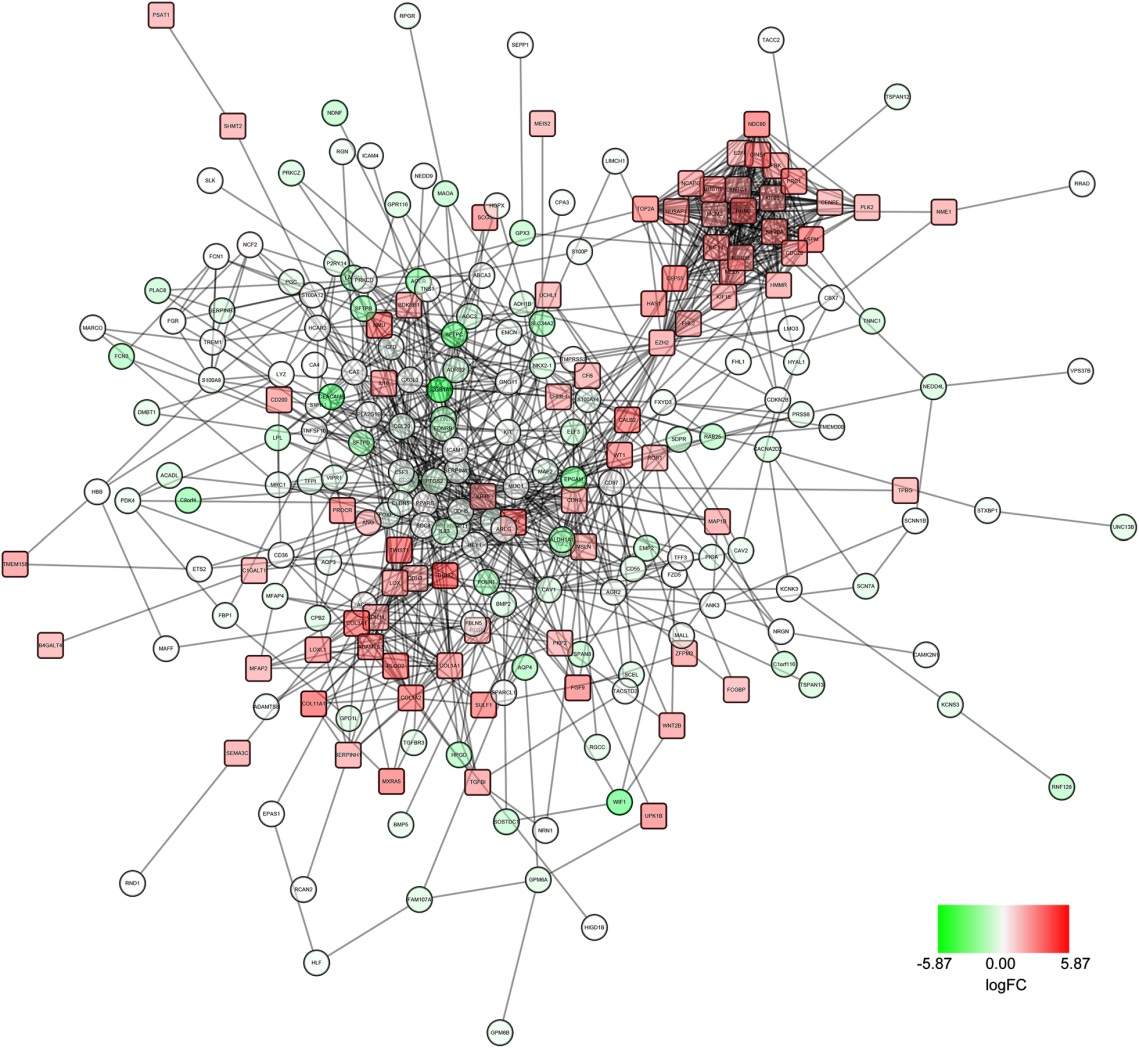
PPI network of the common DEGs constructed using Cytoscape. Rectangles and circles represent up-regulated and down-regulated mRNAs, respectively. The color depth of nodes refers to the log2 (fold-change). *PPI network* protein–protein interaction network, *DEGs* differentially expressed genes

**Fig. 3:**
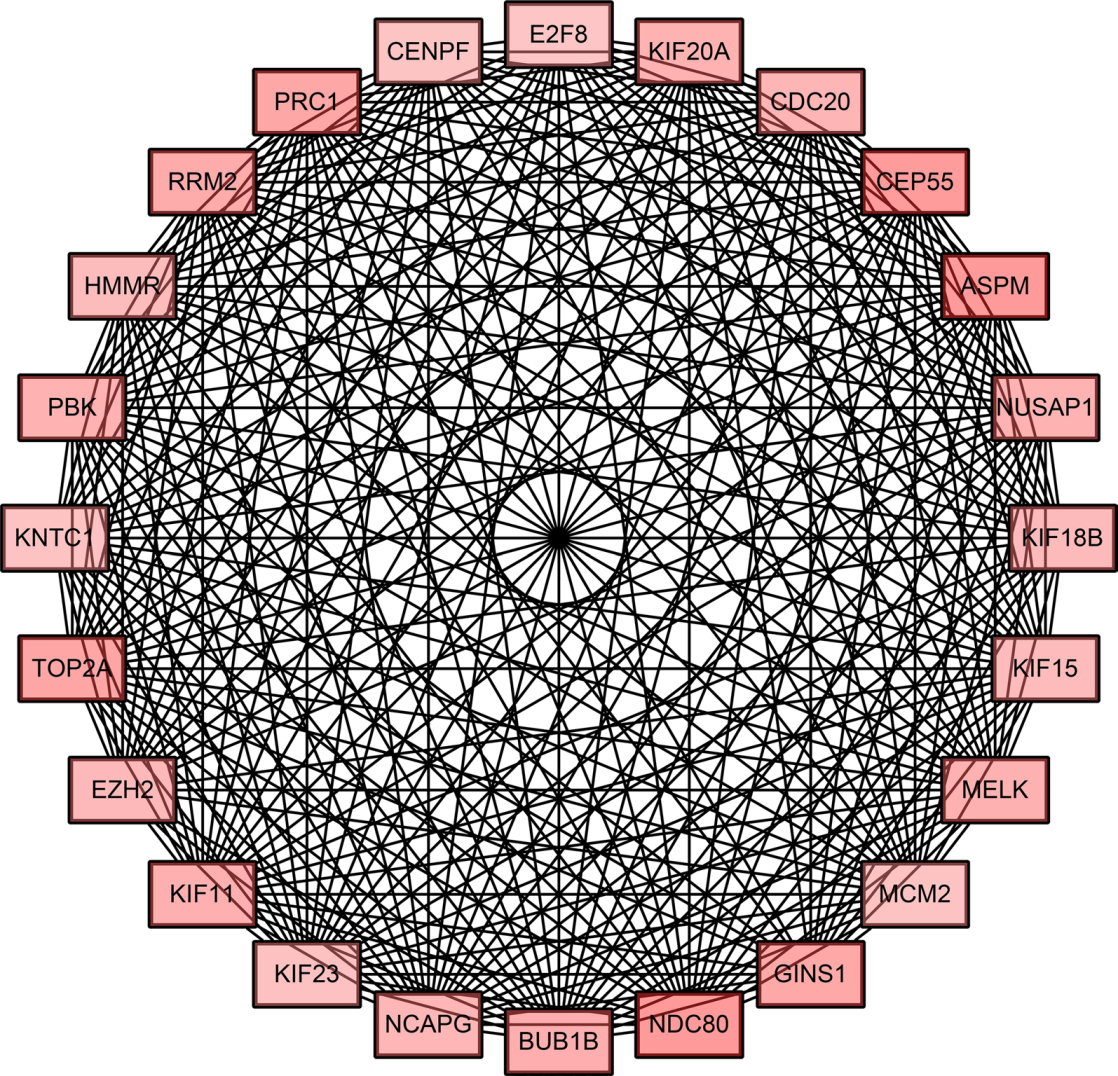
A significant module of hub genes obtained from the PPI network of DEGs using MCODE. *PPI network* protein–protein interaction network, *MCODE* molecular complex detection

To investigate protein interactions, PPI network of the common DEGs was downloaded from STRING and the PPI network was constructed using Cytoscape. The network contained 239 nodes and 56,882 edges after removing isolated nodes (Fig. 2). And then, a module of significant hub genes was obtained from the PPI network of DEGs using MCODE, consisting of 24 nodes and 552 edges (Fig. 3).The hub genes were obtained with a connectivity degree of ≥ 19 in the module. Considering the connectivity and the node status together, EZH2 and HMMR might play the most important roles in the carcinogenesis or progression of MPM (Table 2).

**Table 2:** Characteristics of 24 hub genes obtained from a significant module of the PPI network

Name	Full name	Degree	Regulation	MCODE node status
EZH2	Enhancer Of Zeste 2 Polycomb Repressive Complex 2 Subunit	35	Up	Clustered
KIF11	Kinesin Family Member 11	27	Up	Clustered
ASPM	Abnormal Spindle Microtubule Assembly	26	Up	Clustered
HMMR	Hyaluronan Mediated Motility Receptor	25	Up	Seed
CEP55	Centrosomal Protein 55	25	Up	Clustered
CDC20	Cell Division Cycle 20	25	Up	Clustered
BUB1B	BUB1 Mitotic Checkpoint Serine/Threonine Kinase B	25	Up	Clustered
PRC1	Protein Regulator Of Cytokinesis 1	25	Up	Clustered
RRM2	Ribonucleotide Reductase Regulatory Subunit M2	24	Up	Clustered
TOP2A	DNA Topoisomerase II Alpha	24	Up	Clustered
PBK	PDZ Binding Kinase	24	Up	Clustered
KIF20A	Kinesin Family Member 20A	24	Up	Clustered
MCM2	Minichromosome Maintenance Complex Component 2	24	Up	Clustered
KIF23	Kinesin Family Member 23	24	Up	Clustered
NUSAP1	Nucleolar And Spindle Associated Protein 1	24	Up	Clustered
NCAPG	Non-SMC Condensin I Complex Subunit G	24	Up	Clustered
KIF15	Kinesin Family Member 15	23	Up	Clustered
MELK	Maternal Embryonic Leucine Zipper Kinase	23	Up	Clustered
NDC80	NDC80 Kinetochore Complex Component	23	Up	Clustered
CENPF	Centromere Protein F	22	Up	Clustered
KNTC1	Kinetochore Associated 1	22	Up	Clustered
KIF18B	Kinesin Family Member 18B	21	Up	Clustered
E2F8	E2F Transcription Factor 8	21	Up	Clustered
GINS1	GINS Complex Subunit 1	19	Up	Clustered

*MCODE node status* Molecular Complex Detection Node Status

### GO and KEGG pathway enrichment analysis

**Fig. 4:**
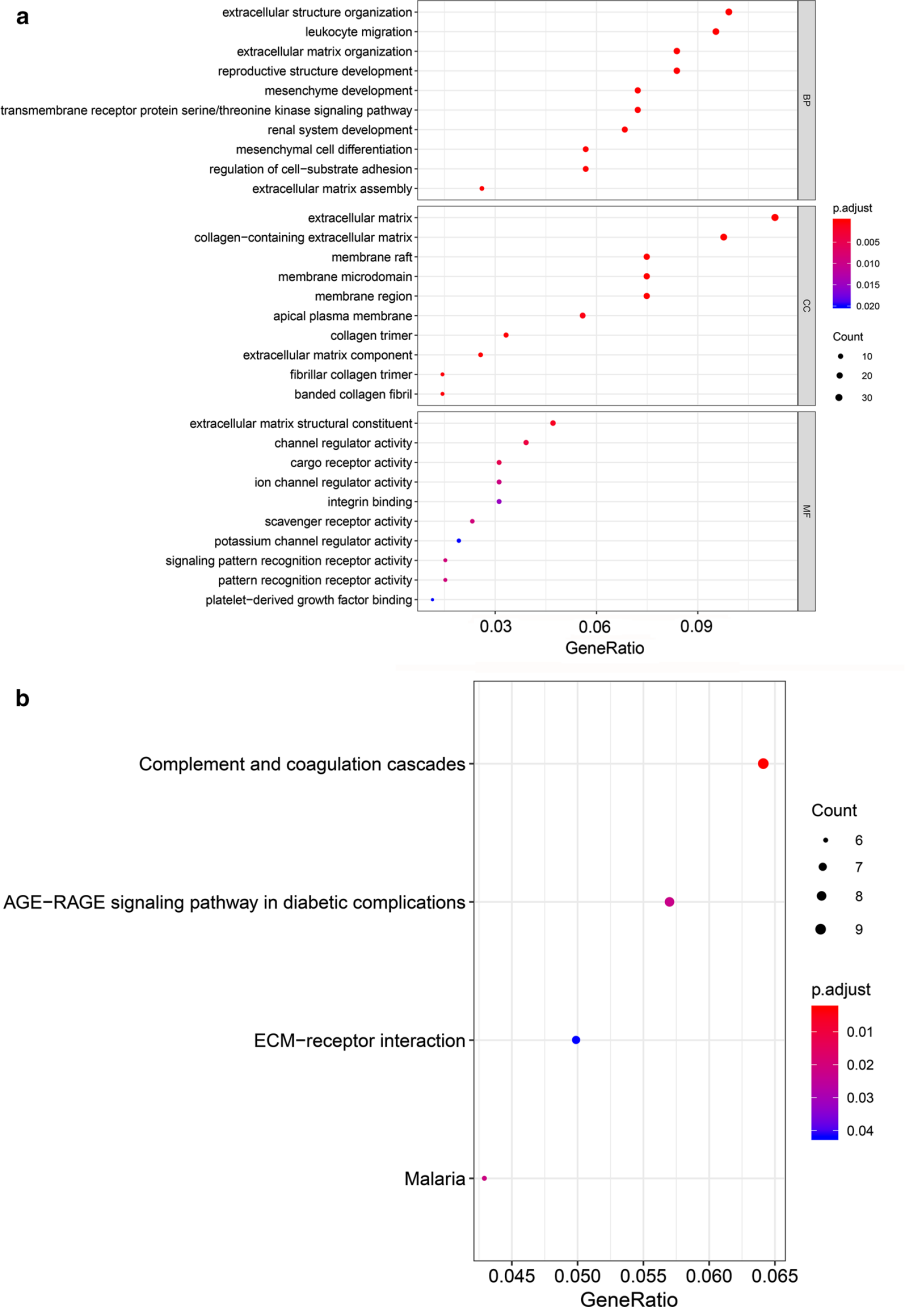
GO and KEGG pathway enrichment analysis of the common DEGs. **a** The top 10 significantly functionally enriched GO terms. Each dot represents a GO term. The dot size represents the count of genes in each term, while colors represent the adjusted P-value. **b** Four mostly enriched KEGG pathways. Each dot represents a KEGG pathway. The dot size represents the count of genes in each term, while colors represent the adjusted P-value. *GO* Gene Ontology, *KEGG* Kyoto Encyclopedia of Genes and Genomes, *DEG* differentially expressed genes, *BP* biological process, *MF* molecular function, *CC* cell component

**Fig. 5:**
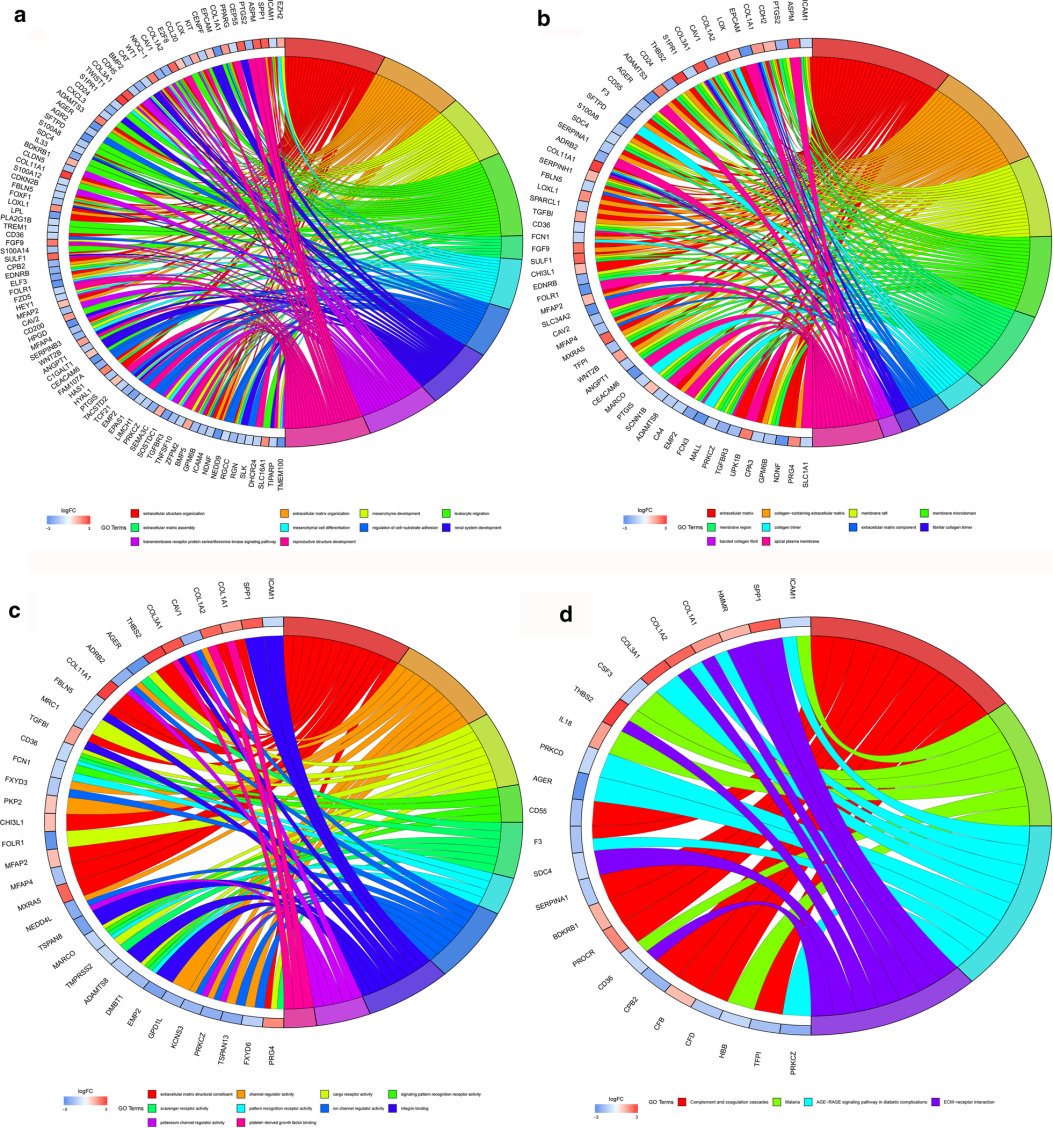
GO and KEGG pathway enrichment analysis for the common genes based on the gene enrichment degree. **a** BP, **b** MF, **c** CC and **d** KEGG pathway analysis. The genes were arranged in circles clockwise direction according to their enrichment degree. The left rectangular colors represent log2 (fold-change) of the common genes. The right rectangular colors represent enriched terms. *GO* Gene Ontology, *KEGG* Kyoto Encyclopedia of Genes and Genomes, *BP* biological process, *MF* molecular function, *CC* cell component

After GO and KEGG pathway enrichment analysis, the top 10 terms of each category ranked by adjusted *P*-value are presented in Fig. 4a. These results showed that the DEGs in biological process (BP) were mainly enriched in extracellular matrix organization, leukocyte migration, extracellular matrix organization, reproductive structure development, mesenchyme development, transmembrane receptor protein serine/threonine kinase signaling pathway, renal system development, mesenchymal cell differentiation, regulation of cell − substrate adhesion and extracellular matrix assembly. The DEGs in cellular component (CC) were mainly enriched in extracellular matrix, collagen − containing extracellular matrix, membrane raft, membrane microdomain, membrane region, apical plasma membrane, collagen trimer, extracellular matrix component, fibrilar collagen trimer, and banded collagen fibril, while the DEGs in molecular function (MF) were significantly enriched in extracellular matrix structural constituent, channel regulator activity, cargo receptor activity, ion channel regulator activity, integrin binding, scavenger receptor activity, potassium channel regulator activity, signaling pattern recognition receptor activity, pattern recognition receptor activity, platelet-derived growth factor binding. KEGG pathway analysis revealed that the the common DEGs were mainly enriched in Complement and coagulation cascades, AGE-RAGE signaling pathway in diabetic complications, ECM–receptor interaction and Malaria (Fig. 4b). The distribution of the common hub genes in the GO and KEGG pathway analysis is shown in Fig. 5. The hub genes in BP, CC and MF were mainly enriched in extracellular structure organization (Fig. 5a), extracellular matrix (Fig. 5b) and extracellular matrix structural constituent (Fig. 5c), respectively. In the KEGG pathways the hub genes were mainly located in ECM − receptor interaction (Fig. 5d).

### Validation of EZH2 and HMMR expression and their prognostic significance

**Fig. 6:**
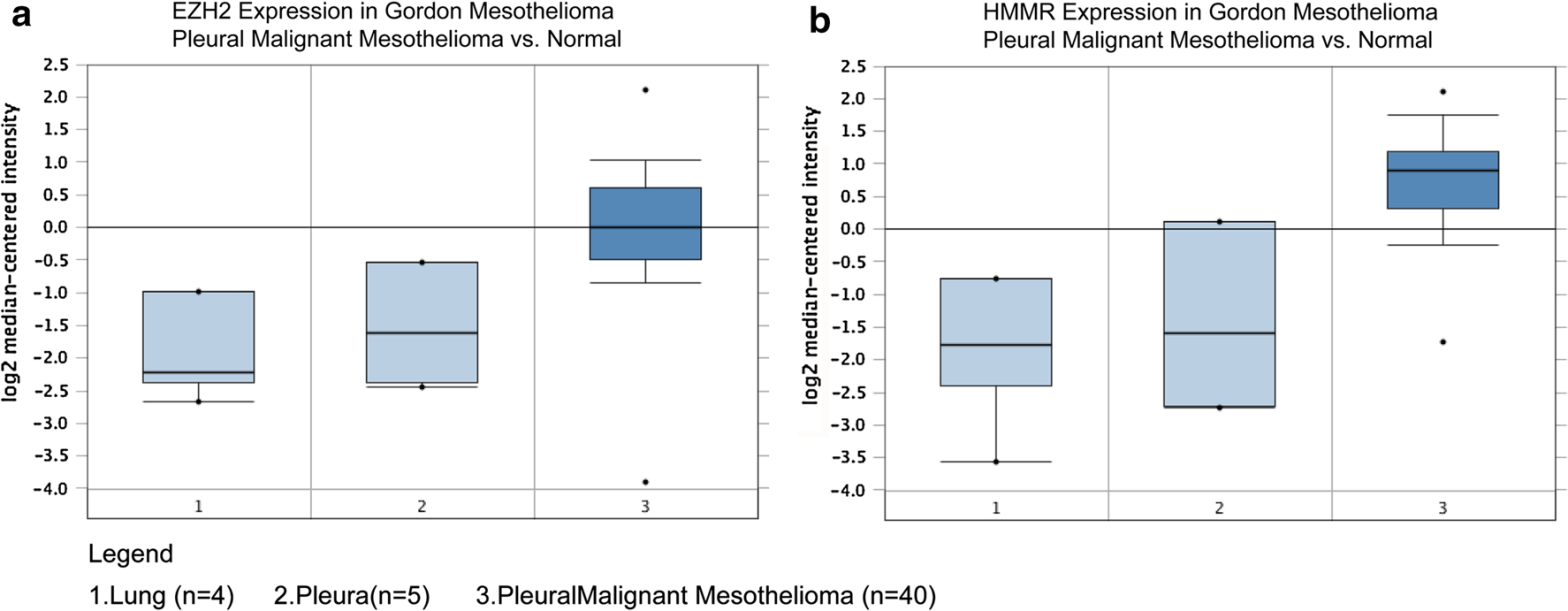
mRNA expression levels of EZH2 (**a**) and HMMR (**b**) in MPM, lung and pleura tissue samples in the ONCOMINE database

**Fig. 7:**
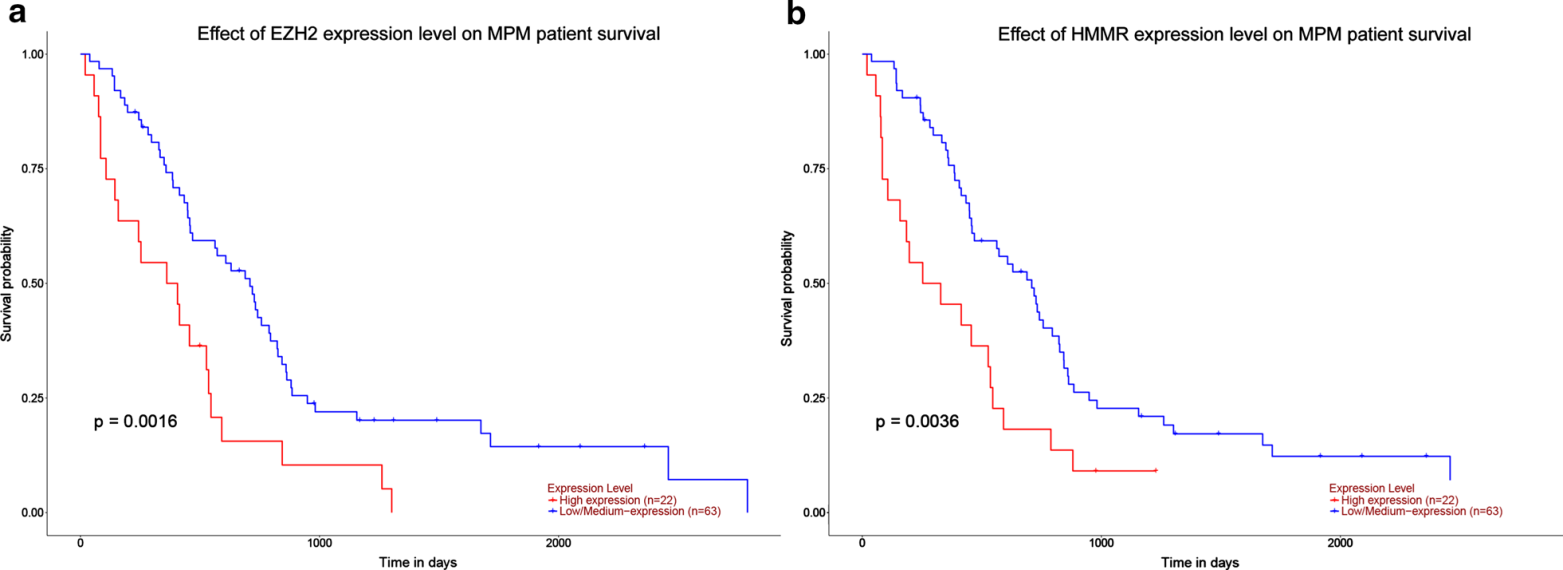
UALCAN overall survival analysis of MPM patients with high and low expression of EZH2 (**a**) and HMMR (**b**)

To validate the expression levels of *EZH2* and *HMMR,* expression data from ONCOMINE were compared. The results showed that they were signifcantly up-regulated in MPM compared to normal tissues (Fig. 6a, b). To investigate the prognostic significance, Kaplan–Meier survival analysis was preformed. The log-rank test confrmed that patients with high levels of EZH2 and HMMR had significantly lower survival rate than those with low levels using UALCAN resource (Fig. 7).

## Discussion

To identify new genes potentially related to MPM, we analyzed two mRNA microarray datasets. A total of 276 common DEGs between MPM tissues and non-cancerous tissues were identifed in the 2 datasets, including 187 downregulated genes and 79 upregulated genes. GO and pathway analyses were performed to explore functions of the DEGs. They were found enriched in extracellular structure organization, extracellular matrix, matrix structural constituent, and ECM–receptor interaction. EZH2 and HMMR were found to be related to MPM based on PPI analysis.

Previous studies have reported that overexpression of HAPLN1, one of the ECM proteins, increases tumorigenic properties of mesothelioma [17]. In addition, high expression of tenascin‐C protein, an extracellular matrix glycoprotein known to have anti‐adhesive characteristics in MPM, might play a role in invasive growth of MPM [18]. KEGG pathway analysis showed that the common DEGs are associated with complement and coagulation cascades, AGE-RAGE signaling pathway in diabetic complications, ECM−receptor interaction, and HMMR was riched in ECM−receptor interaction. Based on parallel sequencing, Hylebos et al. [19] found that tumor protein p53/DNA repair, cell cycle, mitogen-activated protein kinase, and phosphoinisitide 3-kinase (PI3K)/AKT pathways are related to MPM. Furthermore, Cedrés et al. proposed that pS6 might be an independent prognostic factor in MPM [9]. Targeting this pathway could be of therapeutic significance. For instance, in the SWOG S0722 trial mTOR inhibitor everolimus (RAD001) has been demostrated to have limited clinical activity in advanced MPM patients [20].

To examine the associations of protein functions of the identified 276 common DEGs, a PPI network was constructed, 24 DEGs were selected as hub genes because of their high degree of connectivity (> 19) using MCODE in Cytoscape. According to the connectivity degree and the node status, EZH2 and HMMR have signifcant differences between MPM and non-cancerous tissue samples. EZH2 encodes a member of the Polycomb-group (PcG) family in which the two main families are polycomb repressive complex 1 (PRC1) and PRC2 [21]. They are associated with the embryonic ectoderm development protein, the VAV1 oncoprotein, and the X-linked nuclear protein and may play a role in the hematopoietic and central nervous syste [22]. As previously reported, EZH2 is overexpressed in Weaver syndrome and primary cutaneous follicle center lymphoma [22, 23]. LaFave et al. [24] reported that loss of BAP1 promotes MPM cell proliferation by upregulating EZH2. The epigenetic gene H3K27me3 catalyzed by EZH2 is repressive which is aberrantly expressed during malignancy transformation in MPM [25]. Suppression of EZH2 using RNA interference was found to decrease the cancerogeneity of malignant mesothelioma cells [25]. In clinic practice, highly expressed EZH2 and deletion of BAP1 and MTAP can be detected by immunohistochemistry and may be used to distingush MPM from mesothelial hyperplasia [21]. HMMR (Hyaluronan Mediated Motility Receptor) is a protein coding gene influencing in cell motility [26]. The protein is detected in breast tissue and complexes with BRCA1 and BRCA2. And thus, HMMR potentially increases the risk of breast cancer. In addition, EZH2 and HMMR were also overexpressed in MPM from ONCOMINE and discovered to be associated with low overall survival in MPM patients, suggesting that they may be potential diagnostic and prognostic biomarkers for MPM.

There are several limitations in this study. The results and conclusions obtained are based on microarray data, and there is a lack of validation with experimental data with clinical samples. The sample size in prognostic analysis is relatively small. Further biological experiments are needed to elucidate the mechanisms behind the expression changes of these key genes and their biological functions in MPM.

In conclusion, our analysis has identified a number of DEGs that are closely related to the development, progression, and prognosis of MPM. A total of 277 DEGs and 24 hub genes have been found this study. EZH2 and HMMR might be the core genes of MPM based on their high connectivity in the protein interaction network. These findings may provide clues to develop the potential biomarkers for diagnosis of MPM and insight on molecular mechanisms underlying MPM to identify novel pharmacological and therapeutic targets for the treatment of MPM. However, further experimental verifcation and clinical studies are needed to confrm the potential biological functions and prognositic significance for MPM.

## Data Availability

The datasets used and/or analysed during the current study are available from the corresponding author on reasonable request.

## Abbreviaitons

MPM: Malignant pleural mesothelioma
GEO: Gene Expression Omnibus
DEG: Differentially expressed genes
PPI: Protein–protein interaction network
GO: Gene ontology
KEGG: Kyoto encyclopedia of genes and genomes
SETD2: SET domain containing protein 2
STRING: Search Tool for the Retrieval of Interacting Genes
MCODE: Molecular complex detection
MF: Molecular function
CC: Cellular component
BP: Biological process
